# Body composition and body fat distribution in tissue-specific insulin resistance and in response to a 12-week isocaloric dietary macronutrient intervention

**DOI:** 10.1186/s12986-024-00795-y

**Published:** 2024-04-09

**Authors:** Inez Trouwborst, Kelly M. Jardon, Anouk Gijbels, Gabby Hul, Edith J.M. Feskens, Lydia A. Afman, Jennifer Linge, Gijs H. Goossens, Ellen E. Blaak

**Affiliations:** 1 Department of Human Biology, NUTRIM School of Nutrition and Translational Research in Metabolism, Maastricht University Medical Center +, Universiteitssingel 50, 6229 ER Maastricht, the Netherlands; 2 TI Food and Nutrition (TiFN), Wageningen, The Netherlands; 3 Division of Human Nutrition and Health, Wageningen University, Wageningen, The Netherlands; 4 AMRA Medical AB, Linköping, Sweden; 5 Division of Diagnostics and Specialist Medicine, Department of Health, Medicine and Caring Sciences, Linköping University, Linköping, Sweden

**Keywords:** Body composition, Body fat distribution, Whole-body MRI, Whole-body and tissue-specific insulin resistance, Dietary intervention, Isocaloric

## Abstract

**Background:**

Body composition and body fat distribution are important predictors of cardiometabolic diseases. The etiology of cardiometabolic diseases is heterogenous, and partly driven by inter-individual differences in tissue-specific insulin sensitivity.

**Objectives:**

To investigate (1) the associations between body composition and whole-body, liver and muscle insulin sensitivity, and (2) changes in body composition and insulin sensitivity and their relationship after a 12-week isocaloric diet high in mono-unsaturated fatty acids (HMUFA) or a low-fat, high-protein, high-fiber (LFHP) diet.

**Methods:**

This subcohort analysis of the PERSON study includes 93 individuals (53% women, BMI 25–40 kg/m2, 40–75 years) who participated in this randomized intervention study. At baseline and after 12 weeks of following the LFHP, or HMUFA diet, we performed a 7-point oral glucose tolerance test to assess whole-body, liver, and muscle insulin sensitivity, and whole-body magnetic resonance imaging to determine body composition and body fat distribution. Both diets are within the guidelines of healthy nutrition.

**Results:**

At baseline, liver fat content was associated with worse liver insulin sensitivity (β [95%CI]; 0.12 [0.01; 0.22]). Only in women, thigh muscle fat content was inversely related to muscle insulin sensitivity (-0.27 [-0.48; -0.05]). Visceral adipose tissue (VAT) was inversely associated with whole-body, liver, and muscle insulin sensitivity. Both diets decreased VAT, abdominal subcutaneous adipose tissue (aSAT), and liver fat, but not whole-body and tissue-specific insulin sensitivity with no differences between diets. Waist circumference, however, decreased more following the LFHP diet as compared to the HMUFA diet (-3.0 vs. -0.5 cm, respectively). After the LFHP but not HMUFA diet, improvements in body composition were positively associated with improvements in whole-body and liver insulin sensitivity.

**Conclusions:**

Liver and muscle insulin sensitivity are distinctly associated with liver and muscle fat accumulation. Although both LFHP and HMUFA diets improved in body fat, VAT, aSAT, and liver fat, only LFHP-induced improvements in body composition are associated with improved insulin sensitivity.

**Trial registration:**

NCT03708419 (clinicaltrials.gov).

**Supplementary Information:**

The online version contains supplementary material available at 10.1186/s12986-024-00795-y.

## Background

Obesity is accompanied by an increased risk for the development of chronic cardiometabolic diseases, including cardiovascular diseases (CVD), type 2 diabetes (T2D) and several types of cancer. However, individuals with obesity make a heterogeneous group where some develop cardiometabolic diseases while others remain relatively healthy [1, 2]. Furthermore, the etiology towards obesity-associated cardiometabolic diseases is highly heterogenous. Insulin resistance for example can develop to a different extent in insulin-sensitive organs such as the liver and skeletal muscle within individuals with obesity, representing different etiologies towards T2D and cardiometabolic risk [3].

The body mass index (BMI) is a simple and inexpensive measurement that has been extensively used to identify obesity, but it is not a good indicator of cardiometabolic health at the individual level [4]. Body composition and body fat distribution, which include the distribution of fat storage in different adipose tissue depots, skeletal muscle mass, and ectopic fat deposition, can help to explain the differences in cardiometabolic disease risk observed among individuals with overweight or obesity [1, 5–8]. Indeed, excessive abdominal visceral and subcutaneous adipose tissue (VAT and SAT) [6] and low skeletal muscle mass [1, 7], as well as liver fat [6], are strongly associated with whole-body insulin resistance in humans. Body composition and body fat distribution may be important determinants of tissue-specific metabolic disturbances and may thus also be associated with tissue-specific insulin resistance [7, 9]. Notably, clear sex differences in body composition and its relationship to cardiometabolic diseases have been reported, as extensively reviewed [10]. However, whether body composition and body fat distribution can (partially) explain the distinct etiologies of the tissue-specific insulin resistant phenotypes in obesity, and whether this is different between men and women, is unclear.

Adopting a healthy diet is an important strategy for decreasing cardiometabolic disease risk, at least partially due to positive effects on body composition [11–13]. Both quality and quantity of dietary protein, fat and carbohydrate seem to impact body composition, body fat distribution, and ectopic fat deposition [9, 12], as well as affect insulin sensitivity and glucose control [14]. We have recently shown that two isocaloric diets within guidelines of healthy nutrition - a low-fat, high-protein, high-fiber diet (LFHP) and a high mono-unsaturated fatty acid diet (HMUFA) - can both elicit pronounced improvements in body composition and several cardiometabolic parameters [15]. Nevertheless, it is not yet clear what the effect of these two isocaloric healthy diets differing in macronutrient composition are on body composition and body fat distribution, measured with state-of-the-art methodology, including characterization of VAT and SAT, ectopic fat deposition, and skeletal muscle volume and whether these improvements are related to improvements in (whole-body and tissue-specific) insulin sensitivity.

The present study aimed to investigate the relationship between body composition, body fat distribution, ectopic fat deposition, and muscle volume with whole-body and tissue-specific insulin sensitivity. Furthermore, we investigated the impact of a 12-week dietary intervention with either an isocaloric LFHP or a HMUFA diet, both within the context of the Dutch dietary guidelines for healthy nutrition, on changes in body composition and insulin sensitivity, as well as the relationship between improvements in body composition and improvements in insulin sensitivity. The findings may provide leads for dietary intervention strategies that better target cardiometabolic risk factors in obesity.

## Methods

### Study design and population

**Fig. 1:**
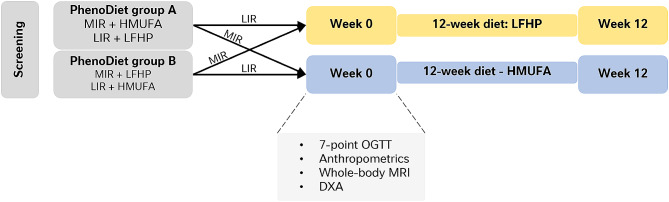
Study design of the subcohort analysis in the present study. Participants were assigned to either a HMUFA (high in mono-unsaturated fatty acids) or a LFHP (low in fat and high in protein and fiber) diet for a duration of 12 weeks. At week 0 and week 12, a 7-point oral glucose tolerance test (OGTT), anthropometric measurements, whole-body MRI, and a DXA-scan were conducted

The present analysis is a part of the larger two-center (Maastricht University Medical Center+ (MUMC+) and Wageningen University (WUR), both The Netherlands) double-blind, randomized dietary intervention trial, the PERSonalized Glucose Optimization Through Nutritional Intervention (PERSON) study. The rationale and methodology of the PERSON study have been described previously [16]. Recruitment started June 2018 and the study was completed November 29, 2021. Originally, 242 participants were included and randomly assigned to either Phenotype Diet (PhenoDiet) group A or PhenoDiet group B. PhenoDiet group A included individuals with MIR following a HMUFA, and individuals with LIR following a LFHP diet. PhenoDiet group B included individuals with muscle (MIR) and liver insulin resistance (LIR) on LFHP and HMUFA diets, respectively. Both researchers and participants were blinded to the participants’ metabolic phenotype (LIR or MIR), and thus blinded to whether participants were allocated to PhenoDiet A or B. The primary outcome of the original study was the change in disposition index, a composite marker of insulin secretion and insulin sensitivity, in PhenoDiet group A vs. B. The presence of tissue-specific insulin resistance was based the glucose and insulin responses from a 7-point oral glucose tolerance test (OGTT) at screening, from which the hepatic insulin resistance index (HIRI) and the muscle insulin sensitivity index (MISI) were calculated [16]. Tertile cut-offs for MISI and HIRI from a previous study (the Maastricht Study [17]) were used to identify individuals with predominant MIR or LIR. Before and after the intervention, extensive measurements in laboratory and daily life were performed. Exclusion criteria for participation included amongst others: not weight stable (> 3 kg weight change in last 3 months), smoking, alcohol abuse (> 14 glasses/week), pre-diagnosis of diabetes, major cardiovascular disease, major gastrointestinal disease or surgery, and dietary restrictions interfering with the dietary study protocol. For the current secondary analysis, the relationship between body composition and body fat distribution with whole-body and tissue-specific insulin sensitivity was determined in 93 individuals from the PERSON study that underwent a whole-body magnetic resonance imaging (MRI) scan at MUMC+. The study design for the secondary analysis is shown in Fig. 1. A comparison was made between participants allocated to follow an isocaloric diet either HMUFA and participants allocated to a LFHP diet for 12 weeks (supplementary Fig. 1).

The study was approved by The Medical Ethics Committee of MUMC+ (NL63768.068.17) and registered at ClinicalTrials.gov (identifier: NCT03708419). All participants provided written informed consent. The study was carried out in accordance with the principles of the Declaration of Helsinki.

### Dietary intervention

The HMUFA diet had a targeted macronutrient composition of 38% of energy from fat (20 en% mono-unsaturated, 8 en% poly-unsaturated, and 8 en% saturated fatty acids), 48% of energy from carbohydrates (30 en% polysaccharides, 3 g/MJ fiber), and 14% of energy from protein. For the LFHP diet, the targeted macronutrient composition was 28% of energy from fat (10 en% mono-unsaturated, 8 en% poly-unsaturated, and 8 en% saturated fatty acids), 48% of energy from carbohydrates (30 en% polysaccharides, > 4 g/MJ fiber), and 24% of energy from protein. Participants received verbal and written dietary instructions at the start of the intervention and received weekly dietary counseling and were provided with key food products. Body weight was monitored every week to ensure participants remained relatively weight stable throughout the intervention. Dietary adherence was assessed with three unannounced one-day food records during the intervention. A detailed description of the dietary compliance has been described elsewhere [16]. In short, the dietary compliance to both the HMUFA as well as LFHP was overall high, with distinct intake between diets in intake from total fat, mono-unsaturated fatty acids, protein, and fiber, and similar intake of saturated fatty acids and total carbohydrates. An extensive description of the details of the dietary intervention are described in detail elsewhere [16].

#### Anthropometrics and body composition

Body weight (kg) and height (cm), and waist and hip circumference (cm) were measured to the closest 0.1 unit in duplicate and in underwear. A subgroup (*n* = 93) of the total PERSON study population (MUMC + participants) underwent a whole body 3T MRI scan (3T MAGNETOM Prisma fit, Siemens Healthcare). Analyses were performed using computational modeling (method by AMRA Medical AB, Linköping, Sweden [18]). Fat ratio: (%, total abdominal adipose tissue / (total abdominal adipose tissue + total thigh muscle volume)*100), VAT volume (L), abdominal subcutaneous adipose (aSAT) volume (L), thigh muscle fat (%), liver fat: (%), abdominal adipose tissue (AT) index (L/m^2^, (VAT + aSAT)/height^2^), weight-to-muscle ratio (kg/L, body weight/total muscle volume), thigh muscle volume (L), and thigh muscle volume Z-score (adjusted for sex and body size (height, body weight, BMI) invariant) were quantified. Participants underwent a dual-energy X-ray absorptiometry (DXA) (MUMC+, Discovery A, Hologic; WUR, Lunar Prodigy, GE Healthcare) to determine body fat %.

### Whole-body and tissue-specific insulin sensitivity

Whole-body and tissue-specific (liver and muscle) insulin sensitivity were estimated based on a 7-point OGTT at baseline and after 12 weeks of intervention. Participants ingested a 200 ml, 75gr glucose solution (Novolab), after an overnight fast. Blood draws were performed from the antecubital vein at t = 0, 15, 30, 45, 60, 90, and 120 min and plasma glucose and insulin were determined. From these glucose and insulin values, we calculated the Matsuda index for whole-body insulin sensitivity, HIRI for liver insulin sensitivity, and MISI for muscle insulin sensitivity. The Matsuda index was calculated by: 10.000 ÷ square root of (fasting plasma glucose (mmol/L) x fasting insulin (pmol/L) x (mean glucose (mmol/L) x mean insulin (pmol/L)) using 5 timepoints of the OGTT (excluding t = 15 and 45). The calculations of the HIRI and MISI have been described previously [16]. Briefly, HIRI was calculated as the area under the curve from both glucose and insulin in the first 30 min of the OGTT while the MISI was calculated by the slope of the peak of the OGTT to the nadir divided by mean insulin concentrations throughout the OGTT. The Matsuda index [19], HIRI, and MISI [20] have been validated against the golden standard hyperinsulinemic clamp technique.

### Statistical analysis

Participant characteristics are presented as mean ± standard deviation (SD) and an independent t-test was performed to assess differences between diets groups, and between sexes at baseline.

#### Cross-sectional relationship between body composition and body fat distribution with (whole-body and tissue-specific) insulin sensitivity

Linear regression analysis was performed to assess crude associations (model 1), with adjustments for age and sex (model 2), additionally adjusted for body weight (model 3), and additionally adjusted for MISI in the model with HIRI and vice versa (model 4). Variables that are expressed relative to body size (for example body fat %) were not adjusted for body weight in model 3 and 4. Effect modification by sex was assessed by addition of an interaction term. In case of significant interaction, results are presented separately for women and men. Data are reported as β (95% confidence interval (CI)).

#### The effects of both diets on changes in (whole-body and tissue-specific) insulin sensitivity, body composition, and body fat distribution

Repeated mixed-model analysis was performed including time (pre- and post-intervention) as repeated measure, while adjusting for age and sex. To examine whether the two diets differentially affected these parameters, an interaction term between diet*time was added. Data are reported as Estimated Marginal Mean (EMM) (95% CI).

#### The associations between diet-induced changes in body composition and changes in (whole-body and tissue-specific) insulin sensitivity

Linear regression analysis was performed including model 1 (with adjustment for diet) is reported, as well as model 2 (additionally adjusted for age, sex, and menopausal status), model 3 (additionally adjusted for weight change (Δ weight)), and model 4 (additionally adjusted for MISI or HIRI in the model with HIRI and MISI as dependent variable, respectively). Interaction terms were included to test for effect modification of diet and sex, and stratified analysis are reported in case of significance. Data are reported as β (95% CI).

Data were transformed with the natural logarithm in case of not-normally distributed residuals. Statistical significance was defined as *P* < 0.05. Analyses were performed using the IBM SPSS Statistics software (version 25).

## Results

### Participant characteristics

Baseline data was available for 94 people (of which 54 were randomized to follow the HMUFA diet, and of which 49 were women) as indicated in Table 1. At baseline, no significant differences were observed between the diet groups, except for waist circumference. Waist circumference was higher in the LFHP group (105.2 ± 9.5 vs. 100.9 ± 10.2, *P* = 0.042), which most likely is explained by the larger, but non-significant, proportion of women in the HMUFA group (57%) compared to LFHP group (46%) (*P* = 0.283). Furthermore, women were slightly younger compared to men (58.3 ± 8.9 vs. 62.0 ± 7.5 years, respectively) (*P* = 0.034). BMI was similar between sexes, on average 31.7 ± 3.8 and 30.8 ± 2.9 kg/m^2^, for women and men, respectively (*P* = 0.231). Parameters of glucose homeostasis were comparable between sexes. On average, individuals were normal glucose tolerant according to clinical cut-off values for fasting and 2-hr glucose values [21]. Large differences between most parameters of body composition were observed between sexes. Women showed greater total body fat %, higher abdominal AT index, and lower VAT volume and muscle fat % compared to men (all *P* < 0.001). Liver fat % and muscle volume Z-score (normalized for sex and body size) were similar between sexes (Table 1).

**Table 1:** Baseline participant characteristics, stratified by diet group and by sex

	HMUFA	LFHP	*P*-value	Women	Men	*P*-value
	(*n* = 54)	(*n* = 39)	diet	(*n* = 49)	(*n* = 44)	sex
**General characteristics**						
Age (years)	59.8 ± 8.8	60.5 ± 7.9	0.667	58.3 ± 8.9	62.0 ± 7.5	**0.034**
Sex (n, % women)	31 (57%)	18 (46%)	0.283	n/a	n/a	
Premenopausal (n, %)	7 (13%)	1 (3%)	0.078	8 (16%)	n/a	
BMI (kg/m^2^)	31.2 ± 3.8	31.4 ± 2.8	0.792	31.7 ± 3.8	30.8 ± 2.9	0.231
Body weight (kg)	90.8 ± 12.7	93.4 ± 12.9	0.336	86.9 ± 11.5	97.4 ± 11.9	**< 0.001**
Waist circumference (cm)	100.9 ± 10.2	105.2 ± 9.5	**0.042**	98.2 ± 9.5	107.7 ± 8.2	**< 0.001**
Waist-to-hip (ratio)	0.92 ± 0.10	0.96 ± 0.09	0.088	0.86 ± 0.06	1.02 ± 0.06	**< 0.001**
Total cholesterol (mmol/L)	5.18 ± 0.91	5.25 ± 0.93	0.698	5.52 ± 0.92	4.86 ± 0.78	**< 0.001**
Systolic blood pressure (mmHg)	126 ± 14	131 ± 11	0.072	123 ± 12	133 ± 12	**< 0.001**
Diastolic blood pressure (mmHg)	83.5 ± 9	85 ± 7	0.317	84 ± 8	85 ± 9	0.700
**Glucose parameters**						
HbA1c (mmol/L)	34.0 [32.0; 37.0]	36.0 [33.0; 40.0]	0.090	34.0 [33.0; 38.5]	34.0 [32.3; 38.0]	0.879
Fasting glucose (mmol/L)	5.5 ± 0.7	5.7 ± 0.7	0.398	5.5 ± 0.7	5.7 ± 0.8	0.187
2-hr glucose (mmol/L)	6.4 [5.1; 7.3]	6.7 [5.2; 8.7]	0.360	6.5 [5.3; 7.3]	6.4 [5.1; 8.5]	0.979
Fasting insulin (pmol/L)	53.4 [37.6; 58.9]	54.5 [38.1; 68.5]	0.356	52.1 [37.2; 66.4]	56.7 [41.1; 67.9]	0.310
HOMA-IR (A.U.)	1.80 [1.26; 2.54]	1.97 [1.46;2.41]	0.302	1.80 [1.18; 2.49]	2.02 [1.37; 2.55]	0.221
Matsuda index (A.U.)	11.2 [7.7; 16.5]	9.2 [6.8; 13.8]	0.284	11.2 [7.3; 17.3]	10.3 [7.0; 13.9]	0.310
HIRI (A.U.)	517 [300; 639]	430 [333; 569]	0.762	418 [308; 588]	423 [311; 599]	0.945
MISI (A.U.)	0.121 [0.088; 0.200]	0.148 [0.100; 0.199]	0.740	0.129 [0.093; 0.199]	0.134 [0.088; 0.209]	0.911
**Body composition**						
Body fat (%)	38.9 [31.1; 44.1]	34.4 [30.9; 43.1]	0.719	43.5 [40.5; 46.0]	30.9 [27.5; 32.6]	**< 0.001**
Fat ratio (%)	58.1 ± 9.4	58.6 ± 7.4	0.758	63.6 ± 6.2	52.3 ± 6.7	**< 0.001**
VAT (L)	5.5 ± 2.3	6.1 ± 2.1	0.209	4.4 ± 1.4	7.3 ± 1.9	**< 0.001**
aSAT (L)	10.0 [6.8; 13.5]	9.8 [8.4; 13.5]	0.933	12.7 [9.9; 14.6]	7.9 [6.4; 9.6]	**< 0.001**
Muscle fat (%)	8.0 [6.2; 9.3]	7.6 [6.7; 8.4]	0.631	8.3 [7.4; 9.8]	6.7 [5.6; 7.7]	**< 0.001**
Liver fat (%)	4.0 [2.7; 9.4]	8.2 [3.6; 16.3]	0.052	4.6 [3.1; 13.6]	5.3 [3.1; 12.2]	0.721
Abdominal AT index (L/m^2^)	5.6 ± 1.6	5.7 ± 1.2	0.750	6.2 ± 1.4	5.0 ± 1.3	**< 0.001**
Weight-to-muscle (ratio)	8.0 [7.0; 9.7]	7.8 [7.1; 9.4]	0.768	9.4 [8.4; 10.3]	7.1 [6.5; 7.5]	**< 0.001**
Muscle volume (L)	11.1 [9.3; 13.4]	12.1 [9.3; 14.4]	0.409	9.3 [8.4; 10.3]	13.9 [12.6; 14.6]	**< 0.001**
Muscle volume (Z-score)	0.224 ± 1.074	0.062 ± 1.055	0.461	0.100 ± 1.148	0.220 ± 0.921	0.584

Data are presented as mean ± standard deviation or median [inter quartile range] in case of non-normally distributed values. An independent sample t-test (normally distributed variables) or a Mann-Whitney U test (non-normally distributed variables) was performed to assess differences between sexes. Significant *P*-values (< 0.05) are highlighted in bold. aSAT, abdominal subcutaneous adipose tissue; HIRI, hepatic insulin resistance index; HOMA-IR, homeostatic model assessment for insulin resistance; MISI, muscle insulin sensitivity index; VAT, visceral adipose tissue

### Associations between body composition and whole-body and tissue-specific insulin sensitivity at baseline

Body weight was significantly associated with the Matsuda index and HIRI, but not with MISI at baseline (Table 2). The Matsuda index, HIRI, and MISI showed distinct associations with parameters of body composition. Only for the association between MISI and muscle fat, a significant sex interaction was present, and data are reported separately for women and men. Specifically, higher muscle fat (%) was associated with lower MISI (i.e., with lower muscle insulin sensitivity) in women (-0.27 (-0.48; -0.05), *P* = 0.016), but not in men. Muscle fat (%) was not related to the Matsuda index or HIRI. Higher liver fat was associated with lower whole-body, liver, and muscle insulin sensitivity (i.e. Matsuda index, HIRI and MISI, respectively), but the significant association between liver fat and MISI disappeared after adjustment for HIRI. All measures of insulin sensitivity were independently associated with the abdominal AT index, VAT, and waist circumference, although the latter two did not reach statistical significance in model 4 (adjustment for age, sex, body weight, HIRI) for MISI (*P* = 0.073 and *P* = 0.063, respectively). Fat ratio (%) was only associated with the Matsuda index (-0.25 (-0.38; -0.13), *P* < 0.001), but not with MISI or HIRI.

**Table 2:** Associations between tissue-specific and whole-body insulin sensitivity and body composition at baseline

				Model 1: Crude		Model 2: Adj. for age and sex		Model 3: Adj. for age, sex, body weight*		Model 4: Adj. for age, sex, body weight*, MISI/HIRI	
				β (95%CI)	*P*-value	β (95%CI)	*P*-value	β (95%CI)	*P*-value	β (95%CI)	*P*-value
**HIRI**	Body weight (kg)			0.17 (0.07; 0.27)	**0.001**	0.21 (0.10; 0.32)	**< 0.001**			0.21 (0.09; 0.33)	**0.001**
	Waist circumference (cm)			0.24 (0.15; 0.34)	**< 0.001**	0.25 (0.15; 0.34)	**< 0.001**	0.27 (0.10; 0.43)	**0.002**	0.22 (0.05; 0.39)	**0.014**
	Body fat (%)			0.00 (-0.11; 0.11)	0.970	0.03 (-0.18; 0.23)	0.795			0.00 (-0.19; 0.20)	0.974
	Fat ratio (%)			0.07 (-0.04; 0.17)	0.230	0.16 (-0.01; 0.33)	0.064			0.10 (-0.04; 0.24)	0.157
	VAT (L)			0.16 (0.06; 0.27)	**0.002**	0.27 (0.14; 0.40)	**< 0.001**	0.18 (0.04; 0.33)	**0.016**	0.16 (0.01; 0.31)	**0.042**
	aSAT (L)			0.06 (-0.08; 0.20)	0.374	0.14 (0.00; 0.28)	0.051	-0.13 (-0.33; 0.07)	0.193	-0.14 (-0.33; 0.06)	0.159
	Muscle fat (%)			0.03 (-0.08; 0.14)	0.599	0.04 (-0.09; 0.16)	0.577	-0.06 (-0.18; 0.07)	0.346	-0.06 (-0.18; 0.06)	0.350
	Liver fat (%)			0.16 (0.05; 0.26)	**0.003**	0.16 (0.05; 0.26)	**0.003**	0.12 (0.01; 0.22)	**0.028**	0.12 (0.01; 0.22)	**0.029**
	Abdominal AT index (L/m^2^)			0.12 (0.01; 0.22)	**0.032**	0.15 (0.04; 0.27)	**0.012**			0.13 (0.01; 0.25)	**0.028**
	Weight-to-muscle (ratio)			0.04 (-0.07; 0.14)	0.500	0.10 (-0.07; 0.26)	0.243			0.07 (-0.09; 0.22)	0.401
	Muscle volume (L)			0.06 (-0.05; 0.17)	0.256	0.21 (0.01; 0.41)	**0.038**	0.05 (-0.17; 0.27)	0.643	0.08 (-0.13; 0.29)	0.471
	Muscle volume (Z-score)			-0.07 (-0.26; 0.11)	0.420	-0.07 (-0.22; 0.07)	0.312			-0.04 (-0.18; 0.10)	0.536
**MISI**	Body weight (kg)			-0.01 (-0.14; 0.12)	0.930	-0.02 (-0.18; 0.14)	0.818			0.07 (-0.09; 0.22)	0.402
	Waist circumference (cm)			-0.10 (-0.23; 0.03)	0.118	-0.14 (-0.29; 0.01)	0.062	-0.30 (-0.52; -0.08)	**0.009**	-0.21 (-0.44; 0.01)	0.063
	Body fat (%)			-0.02 (-0.16; 0.11)	0.725	-0.07 (-0.31; 0.18)	0.591			-0.05 (-0.28; 0.19)	0.700
	Fat ratio (%)			-0.08 (-0.21; 0.06)	0.260	-0.12 (-0.30; 0.05)	0.160			-0.08 (-0.25; 0.09)	0.351
	VAT (L)			-0.10 (-0.23; 0.03)	0.115	-0.18 (-0.35; -0.01)	**0.034**	-0.23 (-0.43; -0.04)	**0.018**	-0.17 (-0.37; 0.02)	0.073
	aSAT (L)			-0.03 (-0.16; 0.11)	0.690	-0.03 (-0.22; 0.13)	0.610	-0.08 (-0.34; 0.19)	0.560	-0.13 (-0.38; 0.12)	0.292
	Muscle fat (%)	Women		-0.18 (-0.38; 0.02)	0.070	-0.20 (-0.42; 0.02)	0.067	-0.19 (-0.42; 0.05)	0.115	-0.27 (-0.48; -0.05)	**0.016**
		Men		0.13 (-0.08; 0.34)	0.228	0.34 (-0.09; 0.34)	0.234	0.11 (-0.13; 0.35)	0.343	0.10 (-0.13; 0.33)	0.396
	Liver fat (%)			-0.14 (-0.27; -0.01)	**0.040**	-0.14 (-0.27; -0.01)	**0.042**	-0.15 (-0.28; -0.01)	**0.041**	-0.09 (-0.23; 0.05)	0.210
	Abdominal AT index (L/m^2^)			0.04 (-0.22; 0.04)	0.166	0.17 (-0.26; 0.03)	0.132			-0.19 (-0.38; 0.00)	**0.045**
	Weight-to-muscle (ratio)			-0.06 (-0.19; 0.07)	0.348	0.35 (-0.32; 0.07)	0.204	0.04 (-0.09; 0.17)	0.498	-0.09 (-0.28; 0.10)	0.342
	Muscle volume (L)			0.50 (-0.12; 0.38)	0.294	0.18 (-0.10; 0.46)	0.205	0.18 (-0.10; 0.46)	0.205	0.19 (-0.08; 0.45)	0.159
	Muscle volume (Z-score)			0.12 (-0.03; 0.26)	0.113	0.11 (-0.05; 0.24)	0.210			0.06 (-0.07; 0.19)	0.340
**Matsuda index**	Body weight (kg)			-0.12 (-0.22; -0.02)	**0.023**	-0.17 (-0.29; -0.06)	**0.004**				
	Waist circumference (cm)			-0.23 (-0.33; -0.14)	**< 0.001**	-0.27 (-0.38; -0.17)	**< 0.001**	-0.33 (-0.49; -0.18)	**< 0.001**		
	Body fat (%)			0.01 (-0.10; 0.11)	0.905	-0.18 (-0.37; 0.01)	0.057				
	Fat ratio (%)			-0.10 (-0.20; 0.00)	0.054	-0.25 (-0.38; -0.13)	**< 0.001**				
	VAT (L)			-0.26 (-0.34; -0.17)	**< 0.001**	-0.36 (-0.47; -0.25)	**< 0.001**	-0.36 (-0.48; -0.23)	**< 0.001**		
	aSAT (L)			-0.03 (-0.14; 0.07)	0.522	-0.16 (-0.28; -0.03)	**0.019**	-0.02 (-0.21; 0.18)	0.862		
	Muscle fat (%)			-0.08 (-0.18; 0.03)	0.135	-0.11 (-0.23; 0.00)	0.058	-0.05 (-0.17; 0.07)	0.382		
	Liver fat (%)			-0.26 (-0.35; -0.17)	**< 0.001**	-0.26 (-0.35; -0.17)	**< 0.001**	-0.24 (-0.33; -0.14)	**< 0.001**		
	Abdominal AT index (L/m^2^)			-0.16 (-0.26; -0.06)	**0.002**	-0.24 (-0.35; -0.14)	**< 0.001**				
	Weight-to-muscle (ratio)			-0.05 (-0.16; 0.05)	0.317	-0.21 (-0.36; -0.07)	**0.005**				
	Muscle volume (L)			-0.03 (-0.13; 0.08)	0.611	0.02 (-0.18; 0.21)	0.847	0.19 (-0.01; 0.4)	0.065		
	Muscle volume (Z-score)			0.11 (-0.02; 0.25)	0.101	0.08 (-0.05; 0.21)	0.227				

* Variables that are expressed relative to body size (body fat, fat ratio, abdominal AT index, weight-to-muscle ratio, muscle volume Z-score) were not adjusted for body weight in model 3 and 4.

Data are reported as β (95% confidence interval (CI)). In case of significant sex interaction, data are reported separately for women and men. Significant *P*-values (< 0.05) are highlighted in bold. Adj, adjusted; aSAT, abdominal subcutaneous adipose tissue; AT, adipose tissue; HIRI, hepatic insulin resistance index; MISI, muscle insulin sensitivity index; VAT, visceral adipose tissue.

### Diet-induced changes in body composition and whole-body and tissue-specific insulin sensitivity

Body weight decreased to a similar extent in both diets, from 90.3 (95% CI, 87.5; 93.3) to 88.4 (85.6; 91.3) kg following HMUFA diet and from 91.3 (88.0; 94.8) to 89.3 (86.0; 92.8) kg following the LFHP diet (time *P* < 0.001, diet x time *P* = 0.824) (Table 3). Body fat, fat ratio, VAT, aSAT, liver fat, the abdominal AT index, and muscle volume also decreased following both diets (all time *P* < 0.05), without significant differences between diets. Waist circumference decreased from 103.8 (101.1; 106.7) to 100.8 (98.2; 103.5) cm following the LFHP diet, while it remained similar (from 100.8 (98.5; 103.1) to 100.3 (98.0; 102.6) cm) following the HMUFA diet (diet x time *P* = 0.006). Trends for greater decrease in muscle fat (diet x time *P* = 0.071), and lower muscle volume (z-score) decrease (diet x time *P* = 0.071) were observed following the LFHP compared to HMUFA diet. The Matsuda index, HIRI, and MISI were not significantly affected by either the HMUFA or LFHP diet (Table 3).

**Table 3:** Changes in parameters of body composition and insulin sensitivity following the HMUFA and LFHP diet

	HMUFA diet (*n* = 40)		LFHP diet (*n* = 30)		*P*-value		
	Week 0	Week 12	Week 0	Week 12	Time	Diet	Diet x time
**Glucose parameters**							
Matsuda index (A.U.)	11.2 (9.8; 12.8)	12.0 (10.5; 13.8)	10.1 (8.6; 11.8)	10.7 (9.1; 12.5)	0.160	0.304	0.895
HIRI (A.U.)	432.9 (375.4; 499.2)	424.4 (358.2; 503.2)	426.0 (360.7; 503.7)	382.3 (313.2; 466.4)	0.777	0.886	0.416
MISI (A.U.)	0.126 (0.106; 0.149)	0.138 (0.116; 0.164)	0.135 (0.111; 0.166)	0.139 (0.114; 0.169)	0.330	0.578	0.635
**Body composition**							
Body weight (kg)	90.3 (87.5; 93.3)	88.4 (85.6; 91.3)	91.3 (88.0; 94.8)	89.3 (86.0; 92.8)	**< 0.001**	0.650	0.824
BMI (kg/m^2^)	29.9 (29.3; 30.5)	29.3 (28.7; 29.9)	29.5 (29.0; 30.1)	28.8 (28.2; 29.3)	**< 0.001**	0.341	0.160
Waist circumference (cm)	100.8 (98.5; 103.1)	100.3 (98.0; 102.6)	103.8 (101.1; 106.7)	100.8 (98.2; 103.5)	0.422	0.101	**0.006**
Body fat (%)	36.1 (35.0; 37.2)	35.2 (34.0; 36.6)	37.3 (36.0; 38.6)	36.4 (34.9; 38.0)	**0.002**	0.177	0.900
Fat ratio (%)	57.6 (55.8; 59.3)	56.6 (54.8; 58.4)	59.5 (57.5; 61.5)	58.1 (56.1; 60.2)	**< 0.001**	0.155	0.212
VAT (L)	5.19 (4.76; 5.65)	4.90 (4.51; 5.33)	5.53 (5.01; 6.11)	5.13 (4.65; 5.65)	**< 0.001**	0.332	0.279
aSAT (L)	9.54 (8.84; 10.3)	9.09 (8.40; 9.84)	10.37 (9.48; 11.34)	9.79 (8.93; 10.74)	**< 0.001**	0.165	0.476
Muscle fat (%)	7.48 (7.08; 7.89)	7.44 (7.05; 7.85)	7.54 (7.08; 8.03)	7.39 (6.94; 7.86)	0.383	0.847	0.071
Liver fat (%)	5.12 (4.08; 6.44)	3.33 (2.62; 4.24)	7.26 (5.53; 9.54)	5.02 (3.77; 6.69)	**< 0.001**	0.055	0.515
Abdominal AT index (L/m^2^)	5.25 (4.92; 5.61)	4.98 (4.65; 5.34)	5.62 (5.20; 6.07)	5.27 (4.86; 5.7)	**< 0.001**	0.196	0.433
Weight-to-muscle (ratio)	8.05 (7.78; 8.33)	7.99 (7.72; 8.26)	8.26 (7.93; 8.60)	8.16 (7.85; 8.49)	**0.012**	0.343	0.515
Muscle volume (L)	11.21 (10.82; 11.61)	11.08 (10.7; 11.47)	11.06 (10.61; 11.52)	10.98 (10.54; 11.43)	**< 0.001**	0.629	0.343
Muscle volume (Z-score)	0.227 (-0.058; 0.511)	0.172 (-0.108; 0.451)	0.027 (-0.304; 0.357)	0.048 (-0.276; 0.37)	0.051	0.366	0.078

Data are presented as Estimated Marginal Mean (EMM) adjusted for age, sex, and study center as analyzed with a mixed model with repeated measure. Significant *p*-values (< 0.05) are highlighted in bold. aSAT, abdominal subcutaneous adipose tissue; AT, adipose tissue; HMUFA, high mono-unsaturated fatty acids; LFHP, low-fat high-protein; VAT, visceral adipose tissue.

### Associations between diet-induced changes in body composition and changes in whole-body and tissue-specific insulin sensitivity

The associations between changes in body composition and changes in (whole-body and tissue-specific) insulin sensitivity upon the dietary intervention are reported in Table 4. Several interactions with diet and with sex were present. In case of these interactions, data are reported for women and men or for both diets separately. In the fully adjusted model, the change in body weight was positively associated with change in HIRI (0.14 (0.05; 0.23), *P* = 0.002) following both diets. Furthermore, the decrease in VAT was associated with a decrease in HIRI, but only following the LFHP diet (0.27 (0.02; 0.52), *P* = 0.039). No associations between change in body weight and VAT were observed with the Matsuda index or with MISI in the fully adjusted models. The change in body fat was associated with the change in MISI only in men in all models (fully adjusted model: -0.11 (-0.21; -0.01), *P* = 0.028). The change in fat ratio following the LFHP diet was negatively associated with the change in Matsuda index, whilst the change in muscle volume was positively associated with the change in Matsuda index, independent of age, sex, and change in body weight. Associations with fat ratio and muscle volume were not observed in relation to the tissue-specific insulin resistance indices HIRI and MISI.

**Table 4:** Associations between the diet-induced changes in tissue-specific insulin resistance and changes in body composition

				Model 1: Adj. for diet		Model 2: Adj. for diet, age, sex, and menopause		Model 3: Adj. for diet, age, sex, menopause and Δ body weight		Model 4: Adj. for diet, age, sex, menopause, Δ body weight, and Δ MISI/HIRI	
				β (95%CI)	*P*-value	β (95%CI)	*P*-value	β (95%CI)	*P*-value	β (95%CI)	*P*-value
Δ **HIRI**	Δ Body weight (kg)			0.16 (0.06; 0.23)	**0.001**	0.15 (0.06; 0.23)	**< 0.001**			0.14 (0.05; 0.23)	**0.003**
	Δ Waist circumference (cm)			0.05 (-0.03; 0.13)	0.241	0.05 (-0.04; 0.13)	0.263	0.00 (-0.08; 0.08)	0.926	0.00 (-0.08; 0.08)	0.985
	Δ Body fat (%)			0.03 (-0.04; 0.11)	0.401	0.03 (-0.05; 0.11)	0.452	-0.01 (-0.09; 0.06)	0.732	-0.02 (-0.10; 0.06)	0.652
	Δ Fat ratio (%)			0.09 (0.02; 0.15)	**0.007**	0.09 (0.02; 0.15)	**0.009**	0.03 (-0.05; 0.11)	0.493	0.03 (-0.05; 0.11)	0.478
	Δ VAT (L)	HMUFA		0.04 (-0.02; 0.11)	0.202	0.05 (-0.02; 0.13)	0.153	0.01 (-0.09; 0.10)	0.873	0.01 (-0.09; 0.11)	0.818
		LFHP		0.27 (0.13; 0.42)	**< 0.001**	0.26 (0.10; 0.43)	**0.003**	0.23 (0.02; 0.45)	**0.037**	0.27 (0.02; 0.52)	**0.039**
	Δ aSAT (L)			0.12 (0.04; 0.19)	**0.002**	0.13 (0.05; 0.21)	**0.002**	0.05 (-0.09; 0.19)	0.494	0.06 (-0.09; 0.21)	0.402
	Δ Muscle fat (%)			0.02 (-0.06; 0.10)	0.656	0.02 (-0.07; 0.10)	0.706	-0.05 (-0.13; 0.04)	0.275	-0.04 (-0.13; 0.04)	0.304
	Δ Liver fat (%)			0.05 (-0.03; 0.13)	0.245	0.05 (-0.03; 0.13)	0.213	0.00 (-0.08; 0.08)	0.967	0.00 (-0.09; 0.08)	0.926
	Δ Abdominal AT index (L/m^2^)			0.12 (0.05; 0.19)	**0.002**	0.13 (0.05; 0.20)	**0.002**	0.05 (-0.08; 0.18)	0.462	0.06 (-0.08; 0.20)	0.413
	Δ Weight-to-muscle (ratio)			0.05 (-0.03; 0.13)	0.181	0.05 (-0.03; 0.14)	0.187	-0.01 (-0.10; 0.08)	0.808	-0.01 (-0.10; 0.08)	0.788
	Δ Muscle volume (L)			0.07 (0.00; 0.15)	0.063	0.08 (0.00; 0.16)	0.056	0.01 (-0.08; 0.10)	0.817	0.02 (-0.08; 0.11)	0.738
	Δ Muscle volume (Z-score)			0.00 (-0.08; 0.08)	0.924	0.00 (-0.08; 0.09)	0.918	-0.01 (-0.08; 0.07)	0.886	0.00 (-0.08; 0.08)	0.983
Δ **MISI**	Δ Body weight (kg)			-0.05 (-0.12; 0.01)	0.108	-0.05 (-0.12; 0.01)	0.103			-0.04 (-0.12; 0.03)	0.244
	Δ Waist circumference (cm)			-0.05 (-0.11; 0.00)	0.060	-0.05 (-0.11; 0.00)	0.068	-0.05 (-0.11; 0.01)	0.123	-0.05 (-0.11; 0.02)	0.131
	Δ Body fat (%)	Women		0.02 (-0.05; 0.08)	0.637	0.01 (-0.05; 0.08)	0.671	0.01 (-0.06; 0.09)	0.715	0.01 (-0.07; 0.09)	0.806
		Men		0.04 (-0.11; 0.20)	0.564	-0.11 (-0.21; -0.02)	**0.020**	-0.11 (-0.20; -0.01)	**0.027**	-0.11 (-0.21; -0.01)	**0.028**
	Δ Fat ratio (%)			-0.03 (-0.08; 0.02)	0.251	-0.28 (-0.08; 0.02)	0.255	-0.01 (-0.08; 0.05)	0.695	-0.01 (-0.08; 0.05)	0.717
	Δ VAT (L)			-0.03 (-0.09; 0.02)	0.248	-0.04 (-0.10; 0.02)	0.153	-0.02 (-0.11; 0.06)	0.568	-0.02 (-0.11; 0.06)	0.614
	Δ aSAT (L)			-0.01 (-0.07; 0.05)	0.741	-0.01 (-0.07; 0.05)	0.749	0.09 (-0.02; 0.19)	0.112	0.09 (-0.02; 0.20)	0.114
	Δ Muscle fat (%)			0.00 (-0.06; 0.06)	0.987	0.00 (-0.06; 0.05)	0.868	0.01 (-0.05; 0.08)	0.723	0.01 (-0.05; 0.08)	0.704
	Δ Liver fat (%)			-0.06 (-0.11; 0.00)	**0.039**	-0.05 (-0.11; 0.03)	0.061	0.05 (-0.11; 0.01)	0.109	-0.05 (-0.11; 0.01)	0.126
	Δ Abdominal AT index (L/m^2^)			-0.03 (-0.09; 0.03)	0.354	-0.03 (-0.09; 0.03)	0.347	0.02 (-0.09; 0.13)	0.700	0.02 (-0.09; 0.13)	0.684
	Δ Weight-to-muscle (ratio)			-0.03 (-0.09; 0.02)	0.235	-0.03 (-0.09; 0.02)	0.257	-0.02 (-0.08; 0.05)	0.589	-0.02 (-0.08; 0.05)	0.633
	Δ Muscle volume (L)			0.01 (-0.04; 0.07)	0.655	0.00 (-0.05; 0.06)	0.880	0.04 (-0.02; 0.11)	0.198	0.04 (-0.03; 0.11)	0.206
	Δ Muscle volume (Z-score)			0.04 (-0.02; 0.10)	0.178	0.03 (-0.02; 0.10)	0.245	0.04 (-0.01; 0.10)	0.138	0.04 (-0.02; 0.10)	0.147
Δ **Matsuda index**	Δ Body weight (kg)			-0.13 (-0.28; 0.01)	0.066	-0.11 (-0.26; 0.03)	0.112				
	Δ Waist circumference (cm)			-0.10 (-0.23; 0.03)	0.130	-0.12 (-0.25; 0.01)	0.063	-0.10 (-0.23; 0.04)	0.153		
	Δ Body fat (%)			-0.07 (-0.20; 0.05)	0.244	-0.12 (-0.24; 0.01)	0.064	-0.09 (-0.22; 0.04)	0.163		
	Δ Fat ratio (%)	HMUFA		-0.09 (-0.18; 0.00)	0.052	-0.06 (-0.15; 0.03)	0.179	0.00 (-0.11; 0.12)	0.937		
		LFHP		-0.21 (-0.43; 0.00)	0.054	-0.25 (-0.48; -0.02)	**0.035**	-0.30 (-0.57; -0.04)	**0.025**		
	Δ VAT (L)			-0.12 (-0.24; 0.01)	0.064	-0.14 (-0.26; -0.02)	**0.022**	-0.13 (-0.3; 0.03)	0.117		
	Δ aSAT (L)			-0.15 (-0.27; -0.02)	**0.022**	-0.13 (-0.26; 0.01)	0.060	-0.11 (-0.33; 0.12)	0.364		
	Δ Muscle fat (%)			-0.08 (-0.21; 0.05)	0.238	-0.11 (-0.24; 0.01)	0.077	-0.08 (-0.22; 0.05)	0.219		
	Δ Liver fat (%)			-0.13 (-0.25; 0.00)	**0.044**	-0.11 (-0.24; 0.01)	0.069	-0.09 (-0.22; 0.05)	0.199		
	Δ Abdominal AT index (L/m^2^)			-0.16 (-0.28; -0.04)	**0.011**	-0.14 (-0.27; -0.02)	0.023	-0.18 (-0.39; 0.04)	0.112		
	Δ Weight-to-muscle (ratio)			-0.12 (-0.25; 0.00)	0.054	-0.10 (-0.22; 0.02)	0.110	-0.07 (-0.21; 0.07)	0.346		
	Δ Muscle volume (L)	HMUFA		-0.10 (-0.22; 0.01)	0.082	-0.07 (-0.19; 0.05)	0.237	-0.01 (-0.14; 0.12)	0.888		
		LFHP		0.18 (-0.07; 0.43)	0.147	0.17 (-0.08; 0.41)	0.172	0.28 (0.01; 0.56)	**0.045**		
	Δ Muscle volume (Z-score)			0.05 (-0.08; 0.18)	0.447	0.06 (-0.07; 0.19)	0.356	0.07 (-0.06; 0.19)	0.277		

Data are reported as β (95% confidence interval (CI)). Δ Indicates the change from week 0 till 12, calculated as value week 12 minus the value of week 0. In case of significant diet or sex interaction, data are reported separately for HMUFA and LFHP or women and men, respectively. Significant *P*-values (< 0.05) are highlighted in bold. Adj, adjusted; aSAT, abdominal subcutaneous adipose tissue; AT, adipose tissue; HIRI, hepatic insulin resistance index; HMUFA, high mono-unsaturated fatty acids; LFHP, low-fat high-protein; MISI, muscle insulin sensitivity index; VAT, visceral adipose tissue.

## Discussion

In this study, we investigated the relationship between body composition and body fat distribution and whole-body and tissue-specific insulin sensitivity in men and women with overweight and obesity using state-of-the-art whole-body MRI technology. Additionally, we investigated diet-induced changes in body composition and the relationship with changes in (tissue-specific) insulin sensitivity after a 12-week isocaloric dietary intervention. We show that impaired liver and muscle insulin sensitivity are associated with distinct body composition profiles. Interestingly, liver fat was associated with impaired liver insulin sensitivity, and in women, an association between muscle fat and impaired muscle insulin sensitivity was found. Furthermore, both the LFHP and HMUFA diet resulted in improvements in body composition in the presence of only minor weight loss (∼2 kg). The LFHP diet resulted in a greater reduction in waist circumferences as compared to the HMUFA diet. The diet-induced changes in whole-body and tissue-specific insulin sensitivity in the current analyses were in line with changes found in the complete study population of the PERSON study [15] but did not reach statistical significance, which seems related to the smaller sample size in the present study. Despite this, the LFHP-induced reduction in VAT was associated with improved liver insulin sensitivity. Furthermore, the decrease in fat ratio and lower decrease in muscle volume were associated with improvements in whole-body insulin sensitivity following the LFHP but not HMUFA diet.

We observed that women had higher total body fat and muscle fat at baseline compared to men, but with similar insulin sensitivity, which is in line with previous studies [10, 22, 23]. This observation may reflect the higher fat storage capacity of women in peripheral tissues including the gluteo-femoral region but also in the skeletal muscle without developing adverse effects on insulin sensitivity. In line, when newly diagnosed with type 2 diabetes, the BMI of women has shown to be almost 2 kg/m^2^ higher despite similar levels of HbA1c [24]. Interestingly, an increase in muscle fat was linked to worse insulin sensitivity in women, but not in men. The underlying mechanisms remain unclear but may related to the fact that women need to attain higher levels of muscle fat to develop insulin resistance, but once women develop prediabetes or T2D, impaired glucose metabolism is more strongly associated with cardiometabolic risk factors compared to men, as reported previously [25]. Notably, most women in the present study were in the postmenopausal state which is associated with decreased estrogen and increased testosterone levels compared to premenopausal women. This, in turn, may impact the association between body composition and metabolic health [22, 26]. It can be speculated that above a certain threshold women may lose their reserve capacity to handle a higher muscle fat storage after menopause. Sexual dimorphism in relation to body composition and cardiometabolic health is well established [1, 27, 28], and confirmed in the present study, but the underlying mechanisms for the present findings remain to be elucidated.

Furthermore, we found that waist circumference and the abdominal AT index were inversely associated with whole-body, as well as tissue-specific insulin sensitivity. Waist circumference is often used as an indicator of abdominal adipose tissue mass [29]. Adipose tissue within the abdominal region, including both aSAT and VAT, has been linked to an adverse cardiometabolic health profile in previous studies [30, 31]. The measurement of waist circumference in the present study thus was a good indicator for overall abdominal-obesity related cardiometabolic risk (i.e. insulin resistance). Nevertheless, determining waist circumference does not allow for discrimination between abdominal SAT and VAT [32]. With more detailed characterization of body composition, as done in the present study, we were able to demonstrate that the associations between abdominal adiposity and whole-body and tissue-specific insulin resistance appeared to be mainly driven by VAT volume, rather than aSAT volume. In line with this, VAT has been previously reported to be linked to an even more adverse metabolic, inflammatory, and dyslipidemic phenotype compared to aSAT [30, 33]. It should be noted however that VAT may be a result of spillover of other AT depots, such as the aSAT, resulting from dysfunctional adipose tissue which fails to appropriately expand [1, 34].

Besides cross-sectional associations, we found improvements in body composition following either a 12-week LFHP or HMUFA diet, in the presence of minor weight loss (∼2 kg). Interestingly, the LFHP diet demonstrated more pronounced improvements in waist circumference, a tendency towards a higher reduction in muscle fat, and lower reduction in muscle volume (Z-score). Previous studies also reported beneficial effects of a low-fat diet on body composition [35, 36]. For example, the LIPGENE study investigated the effect of diets differing in dietary fat quantity and quality on metabolic health in individuals with the metabolic syndrome during a 12-wk period, showing that two isocaloric low-fat diets (28 en% fat) resulted in greater reduction in body weight compared to isocaloric higher-fat diets (38 en% fat) [35]. In another study, body weight and waist circumference decreased more following a low-fat (28 en%) versus a Mediterranean diet rich in MUFA’s without energy restrictions for 2 years [36]. The higher dietary fiber content [37, 38] and higher protein content [39] in the LFHP diet in the present study may also have contributed to the more beneficial effects on body composition. Despite the lack of significant improvements in insulin sensitivity in this subgroup that participated in the PERSON study, we found that LFHP diet-induced reductions in VAT were associated with improvement in HIRI. Additionally, the LFHP-induced decrease in fat ratio and lower reduction of muscle volume were associated with improvements in whole-body insulin sensitivity, whereas this was not seen following the HMUFA diet. To the best of our knowledge, the present study is one of the first studies to show that LFHP-, but not HMUFA-induced improvements in body composition are associated with improvements in whole-body and liver insulin sensitivity.

One of the strengths of the present study is the very well-detailed analysis of body composition and body fat distribution. Body composition can be measured with high accuracy and precision with a whole-body MRI, and this technique may even be considered the gold standard to measure body fat distribution [40]. Nevertheless, this study also has some limitations. The association between body composition and insulin sensitivity might be different for women in the premenopausal compared to postmenopausal state. Due to the relatively small number of premenopausal women in the present analysis (*n* = 8), we were not able to confirm this. Addition of the premenopausal status as a covariate in the analysis did not alter the conclusions of the present study. Future studies are warranted to investigate the relationship between body composition and insulin sensitivity in premenopausal women and age-matched men. Furthermore, we did not observe significant improvements in the Matsuda index, HIRI, and MISI following dietary intervention on a group level, as a result of limited power in this subset, since improvements were observed in the complete PERSON study population [15]. Despite this, we were still able to identify significant associations between changes in body composition and changes in insulin sensitivity. Lastly, it is important to acknowledge that the current analysis predominantly focused on Caucasian older adults (> 50 years), and as a result, the findings cannot be extrapolated to other age groups or ethnicities.

## Conclusion

In summary, tissue-specific insulin resistant phenotypes are associated with distinct body composition profiles. Specifically, body weight and liver fat were associated with liver insulin sensitivity while muscle fat was associated with muscle insulin sensitivity in women but not in men. VAT was independently associated with both whole-body and tissue specific insulin sensitivity. Distinct phenotypes of body composition have previously been identified in individuals with similar BMI, possibly representing different etiologies towards T2D or CVD [5, 18, 41]. Our findings support the presence of distinct metabolic profiles depending on the tissue site of insulin resistance (either in the liver or muscle), which is in line with previous research which identified distinct lipidome, metabolome, and inflammatory profiles in individuals with liver versus muscle insulin resistance [42–44]. Furthermore, we observed that body composition and body fat distribution improved following isocaloric 12-week healthy diets, in the presence of only minor weight loss. Although both the LFHP and HMUFA diet elicited beneficial effects on body composition and body fat distribution, the LFHP appeared most effective in reducing waist circumference. Additionally, only LFHP-induced improvements in body composition were associated with improved (whole-body and liver) insulin sensitivity. Findings of this study give more insight into the heterogeneity of the etiology towards cardiometabolic diseases and can have implications for the development of more targeted dietary intervention strategies to improve cardiometabolic health.

### Electronic supplementary material

Below is the link to the electronic supplementary material.

Supplementary Material 1

## Data Availability

Data is available upon reasonable request from the corresponding author.
