# Supplementary figures and images for: Body composition and body fat distribution in tissue-specific insulin resistance and in response to a 12-week isocaloric dietary macronutrient intervention
Source: Nutr Metab (Lond). 2024 Apr 9;21:20. doi: 10.1186/s12986-024-00795-y (PMC11003022; doi:10.1186/s12986-024-00795-y)

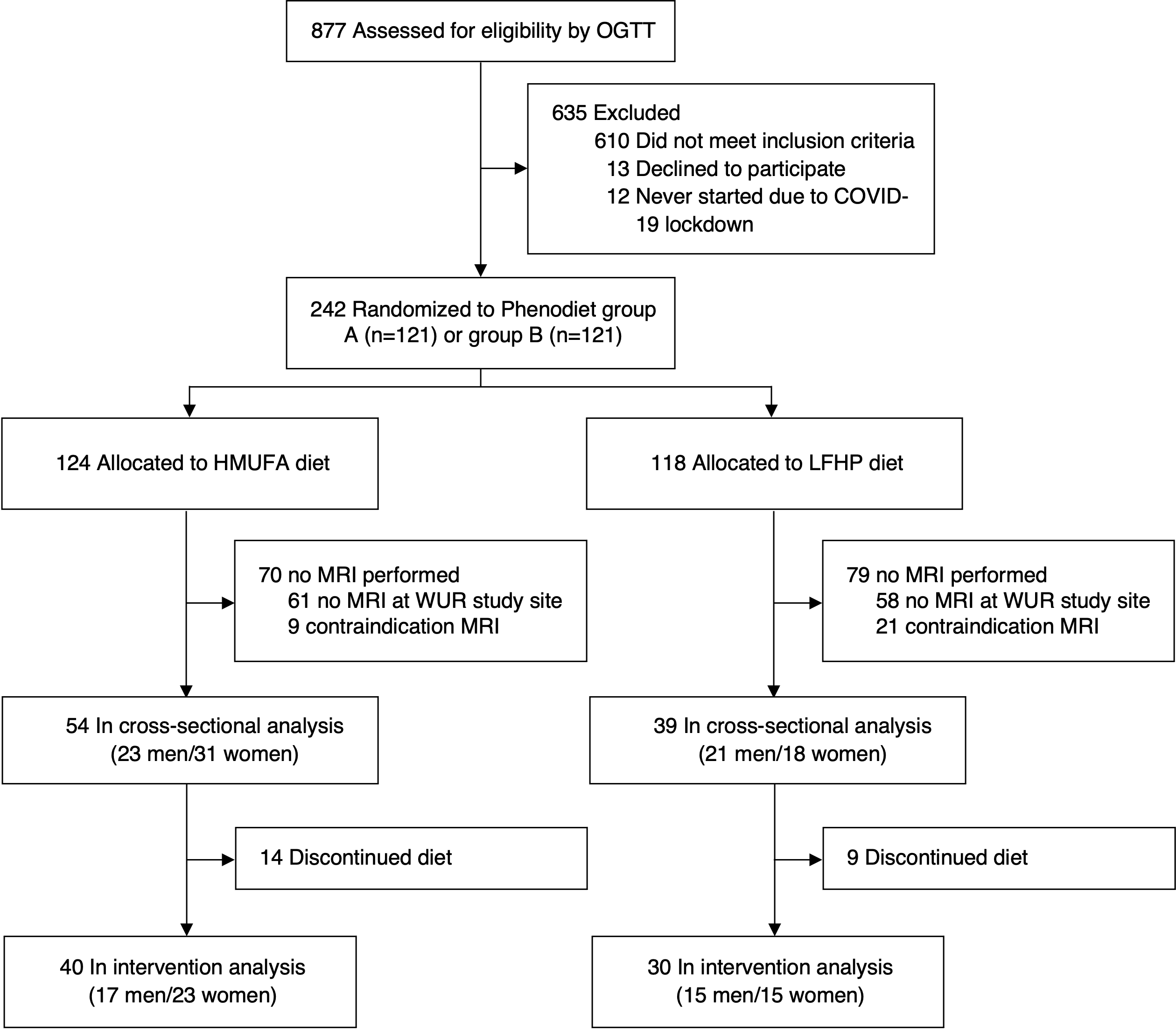

Supplement: Supplementary file 1 — Supplementary Material 1 [file 12986_2024_795_MOESM1_ESM.png]
